# Cearoin Induces Autophagy, ERK Activation and Apoptosis via ROS Generation in SH-SY5Y Neuroblastoma Cells

**DOI:** 10.3390/molecules22020242

**Published:** 2017-02-06

**Authors:** Tonking Bastola, Ren-bo An, Youn-Chul Kim, Jaehyo Kim, Jungwon Seo

**Affiliations:** 1 Institute of Pharmaceutical Research and Development, College of Pharmacy, Wonkwang University, Iksan 570-749, Korea; tonkingbastola@gmail.com (T.B.); anrb@ybu.edu.cn (R.A.); yckim@wku.ac.kr (Y.-C.K.); 2 Hanbang Body-Fluid Research Center, Wonkwang University, Iksan 570-749, Korea; 3 Laboratory of Natural Resources of Changbai Mountain & Functional Molecules Yanbian University, Ministry of Education, Yanji 133002, Jilin, China; 4 Department of Meridian & Acupoint, College of Korean Medicine, Wonkwang University, Iksan 570-749, Korea

**Keywords:** neuroblastoma, cearoin, apoptosis, autophagy, reactive oxygen species, ERK

## Abstract

Neuroblastomas are the most common solid extracranial tumors in childhood. We investigated the anticancer effect of cearoin isolated from *Dalbergia odorifera* in human neuroblastoma SH-SY5Y cells. SH-SY5Y cells were treated with various doses of cearoin. The viability was measured by MTT assay. DCFDA fluorescence assay and Griess assay were used for the measurement of intracellular reactive oxygen species (ROS) and nitric oxide (NO), respectively. Western blot analysis was performed to clarify the molecular pathway involved. Cearoin induced cell death in a dose-dependent manner. Cearoin increased the phosporylation of ERK, the conversion of LC3B-I to LC3B-II, decrease in Bcl2 expression, the activation of caspase-3, and the cleavage of PARP, indicating the induction of autophagy and apoptosis. Furthermore, cearoin treatment increased the production of ROS and NO. Co-treatment with the antioxidant *N*-acetylcysteine completely abolished cearoin-mediated autophagy, ERK activation and apoptosis, suggesting the critical role of ROS in cearoin-induced anticancer effects. Moreover, co-treatment with ERK inhibitor PD98059 partially reversed cearoin-induced cell death, indicating the involvement of ERK in cearoin anticancer effects. These data reveal that cearoin induces autophagy, ERK activation and apoptosis in neuroblastoma SH-SY5Y cells, which is mediated primarily by ROS generation, suggesting its therapeutic application for the treatment of neuroblastomas.

## 1. Introduction

Neuroblastomas are tumors that develop from immature nerve cells of the sympathetic nervous system of infants. Neuroblastomas are the most common solid extracranial neoplasms that occur in childhood, accounting for about 10% of all cancers in children. About 1 out of 3 neuroblastomas develop in the adrenal gland, 1 out of 4 in postganglionic sympathetic nerves in the abdomen and the rest initiate in sympathetic ganglia near the spine in the chest, neck, or pelvis. They account for 6% of all cancers in children aged up to 14 years, and about 700 new cases of neuroblastoma are observed each year in United States [1,2]. Depending on the stage, the treatment of neuroblastoma proceeds, which may include surgery, chemotherapy, radiation therapy, stem cell transplant, retinoid therapy and immunotherapy. The heterogeneous nature of neuroblastoma in a morphological and biochemical context, along with the alteration in karyotype and cytogenetic characteristics helps these tumors to resist the available therapies [3]. Therefore, the risk remains constant for the patients. It is necessary to find novel therapeutic compounds that can work broadly, effectively and efficiently against neuroblastomas.

Natural products play an important role in the research to develop treatment drugs for various diseases. Over 60% of approved and pre-FDA (Food and Drug Administration) candidates are either natural products or natural product-related agents, excepting antibodies and vaccines [4]. In case of anticancer agents, about 75% of anticancer compounds used in medicine are of natural product origin [5]. Anticancer compounds belong to various structural classes such as anthracyclines, enediynes, indolocarbazoles, isoprenoids, polyketides, and non-ribosomal peptides. They induce apoptosis by several mechanisms such as DNA cleavage through the inhibition of topoisomerases, mitochondrial permeabilization, cellular metabolism inhibition, or the inhibition of signal transduction pathways [6].

Cearoin, (2,5-dihydroxy-4-methoxyphenyl)-phenylmethanone, is a benzophenone isolated from the heartwood of *Dalbergia odorifera*. The *Dalbergia odorifera* is a medicinally important plant mainly found in China. Traditionally heartwood is used for treating blood disorders, ischemia, swelling and rheumatic pain in China and Korea. Recently, it was reported that cearoin isolated from *Nepalese propolis* inhibited inflammatory responses in murine macrophagy RAW264.7 cell line [7] and bone marrow-derived mast cells [8]. Cearoin decreased LPS-induced activation of nuclear factor κB (NF-κB), a critical transcription factor for the mRNA expression of several inflammatory mediators in RAW 264.7 cells. Cearoin suppressed nitric oxide (NO) production through inhibiting iNOS mRNA expression and decreased the mRNA expression of TNFα and CCL-2, which were mediated by the inhibition of NF-κB activity. In addition, cearoin inhibited IL-33-induced activation of NF-κB through the suppression of IKK activation, thereby decreasing the mRNA expression of IL-6, TNFα and IL-13 in bone marrow-derived mast cells. From these recent reports, the anti-inflammatory effects of cearoin were elucidated. However, other pharmacological effects of cearoin remain obscure. In particular, the effects of cearoin in human neuroblastoma cells have not been reported yet. In this study, we investigated the anticancer effects of cearoin and its underlying mechanism in human neuroblastoma SH-SY5Y cells. We demonstrate for the first time that cearoin increases autophagy and apoptosis through the production of reactive oxygen species (ROS) and the activation of ERK.

## 2. Results

### 2.1. Cearoin Decreases Cell Viability in SH-SY5Y Neuroblastoma Cells

**Figure 1:**
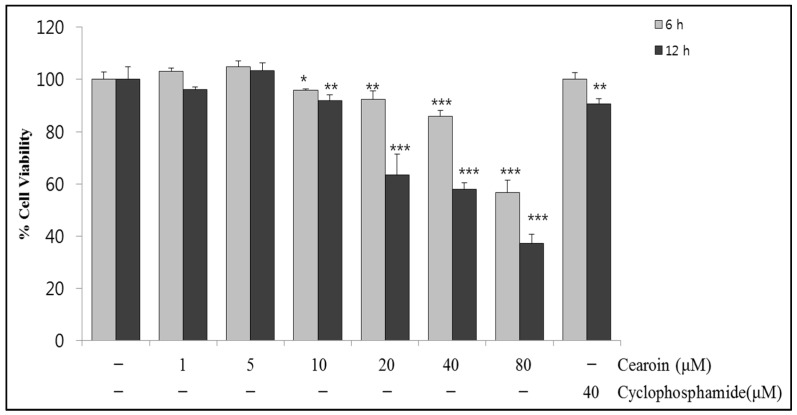
Cearoin reduces viability of human neuroblastoma SH-SY5Y cells. SH-SY5Y neuroblastoma cells were treated with cearoin in various concentrations (0, 1, 5, 10, 20, 40, or 80 μM) and with 40 μM cyclophosphamide as positive control and incubated for 6 h or 12 h. The cell viability was measured using MTT assay. Each bar represents the mean percentage alterations below control (±SD) (*n* = 5~6). Differences were statistically significant at * *p* < 0.05, ** *p* < 0.01 and *** *p* < 0.001 as compared with the control.

Initially, to investigate the cytotoxic effects of cearoin in neuroblastomas, SH-SY5Y cells were treated with various concentrations of cearoin and also with a common anticancer drug, cyclophosphamide, as a positive control in 40 μM concentration for 6 h or 12 h. MTT assay showed that cearoin significantly decreased cell viability from 10 μM in a dose-dependent manner. Treatment with 40 μM cearoin for 12 h induced about 50% loss in cell viability in SH-SY5Y cells, whereas cyclophosphamide induced about 10% loss in cell viability at 12 h (Figure 1). This data suggests the anticancer effects of cearoin.

### 2.2. Cearoin Induces ERK Phosphorylation and Autophagy in SH-SY5Y Cells

**Figure 2:**
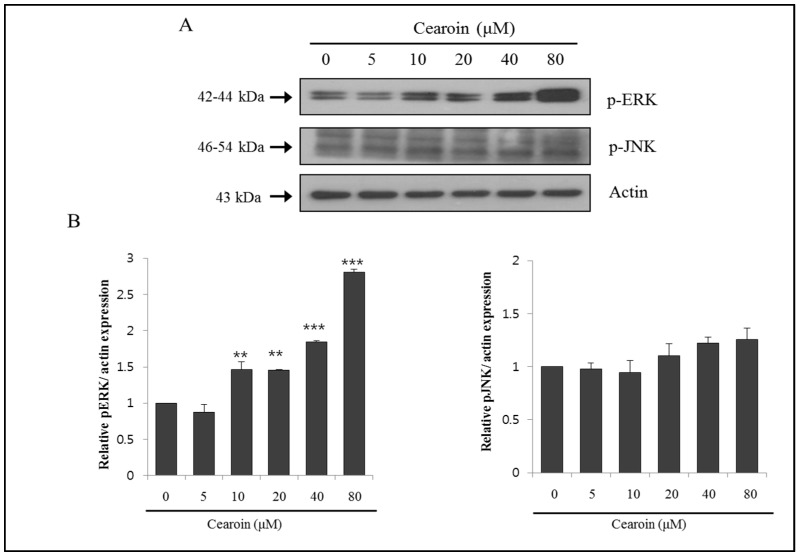

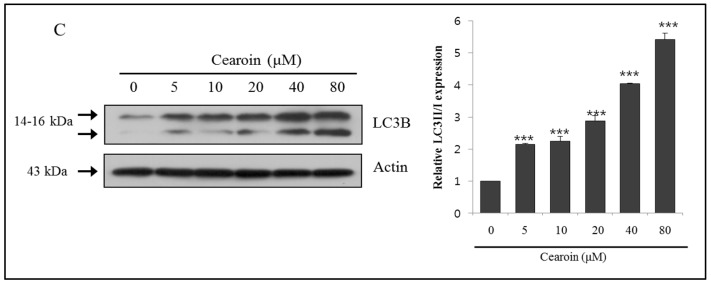
Cearoin induces the phosphorylation of ERK and the formation of LC3B-II. SH-SY5Y neuroblastoma cells were treated with cearoin in various concentrations (0, 5, 10, 20, 40, or 80 μM) and incubated for 12 h. Then the cells were lysed, and (**A**) the expression levels of p-ERK, p-JNK, actin; (**C**) LC3B I/II and actin were measured by Western blot analysis. The blots shown are representative of three independent experiments. Actin was used as a loading control; (**B**) quantification by densitometry. Each bar represents the mean fold alterations above control (±SD) (*n* = 3). Differences were considered statistically significant at ** p < 0.01 and *** p < 0.001 as compared with the control.

Next we examined cearoin-induced intracellular signaling transduction. Western blot analysis showed that cearoin increased ERK phosphorylation in a dose-dependent manner, whereas it did not alter JNK phosphorylation (Figure 2A). During the autophagosome formation, LC3B-I is converted into LC3B-II by conjugating with phosphatidyl ethanolamine. Therefore, the expression of LC3B-II is a good marker for autophagosome formation in the autophagy process [9]. The cearoin in SH-SY5Y cells induced the formation of LC3B-II in a dose dependent manner (Figure 2C), suggesting that cearoin induces autophagy.

### 2.3. Cearoin Induces Apoptosis in SH-SY5Y Cells

**Figure 3:**
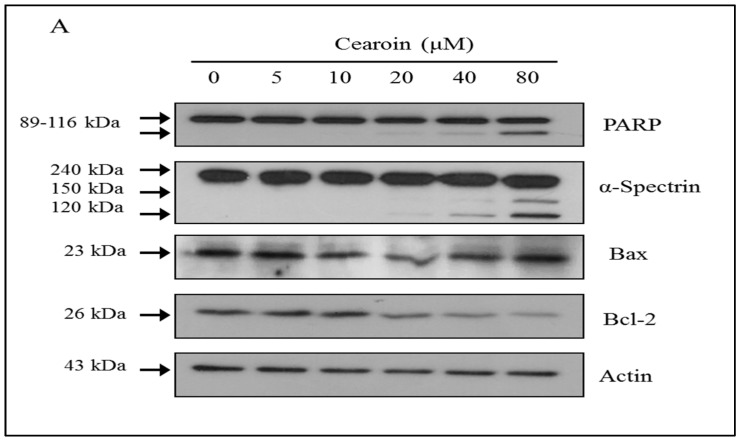

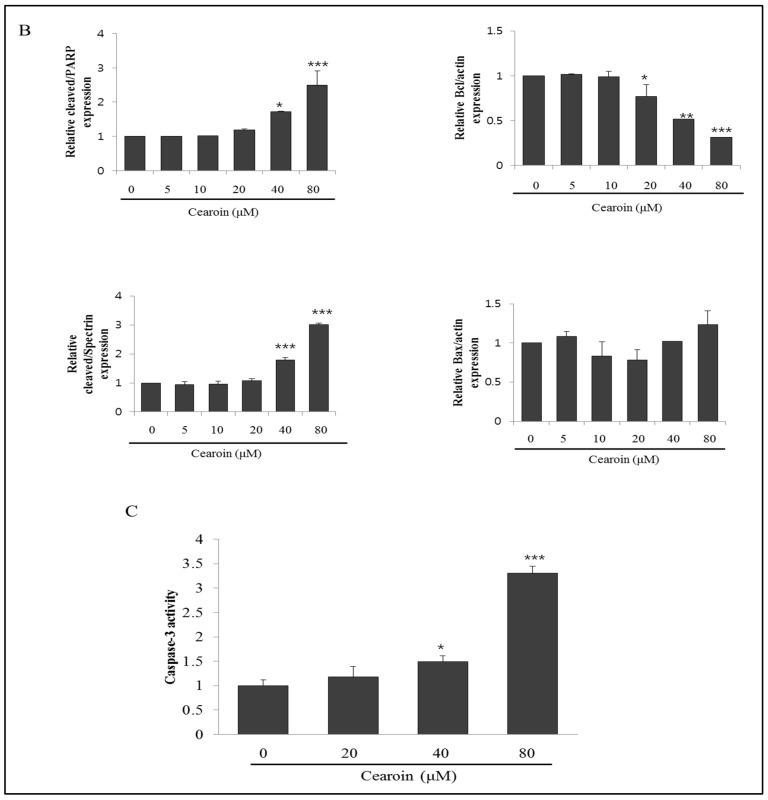
Cearoin induces apoptotic cell death in SH-SY5Y neuroblastoma cells. (**A**) SH-SY5Y neuroblastoma cells were treated with cearoin in various concentrations (0, 5, 10, 20, 40, or 80 μM) and incubated for 12 h. Then, the cells were lysed, and the proteins levels of PARP, α-spectrin, Bax, Bcl-2 and actin were determined by Western blot analysis. The blots shown are representative of three independent experiments. Actin was used as a loading control; (**B**) quantification by densitometry. Each bar represents the mean fold alterations above the control (±SD) (*n* = 3); (**C**) SH-SY5Y neuroblastoma cells were treated with cearoin in various concentrations (0, 20, 40, or 80 μM) for 3 h and the caspase-3 activity was measured using caspase-3 activity assay kit. Each bar represents the mean fold alterations above the control (±SD) (*n* = 3). Differences were considered statistically significant at * *p* < 0.05, ** *p* < 0.01 and *** *p* < 0.001 as compared with the control.

Western blot analysis showed that 40 or 80 μM of cearoin clearly increased PARP cleavage, which indicates caspases activation and the progress of apoptotic cell death. When caspases and/or calpains are activated, the expression of α-spectrin breakdown products (SBDP) is shown at 150 kDa by calpain activation and further cleaved by caspase-3 yielding 120 kDa of shorter fragments. The treatment of 40 μM of cearoin increased caspase-3 mediated SBDP and the treatment of 80 μM of cearoin increased the expression of both calpain and caspase-3 mediated SBDP (Figure 3A). The pro-apoptotic enzyme Bax and the anti-apoptotic enzyme Bcl-2 play crucial roles in regulating cell death and survival [10,11]. Interestingly, the expression of Bax was not altered by cearoin treatment, whereas that of Bcl-2 was significantly decreased in a dose-dependent manner (Figure 3A). In addition, cearoin treatment for 3 h significantly increased the activity of caspase-3 which is activated during apoptosis and used as an apoptotic marker (Figure 3C). These data suggest that cearoin leads to apoptotic cell death in SH-SY5Y cells.

### 2.4. Cearoin Increases the Generation of ROS and NO

**Figure 4:**
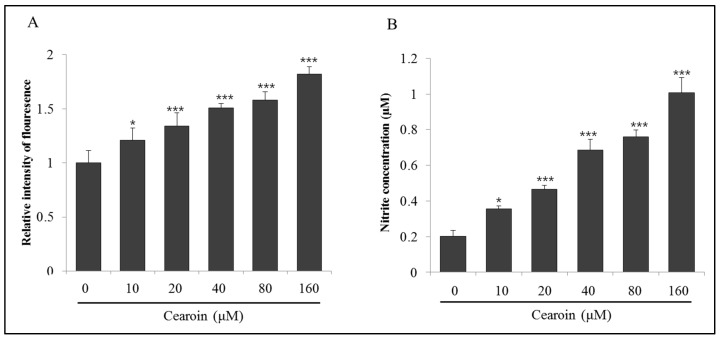
Cearoin increases the intracellular reactive oxygen species (ROS) production and nitric oxide (NO) generation in neuroblastoma SH-SY5Y cells. SH-SY5Y neuroblastoma cells were treated with cearoin in various concentrations (0, 10, 20, 40, 80, or 160 μM) and incubated for 12 h. (**A**) Intracellular ROS production was measured using DCFDA fluorescence assay. Each bar represents the mean percentage alterations above the control (±SD) (*n* = 5); (**B**) total NO release was measured using Griess method. Each bar represents the mean nitrite concentration (±SD) (*n* = 6). Differences were considered statistically significant at * *p* < 0.05 and *** *p* < 0.001 as compared with the control.

Intracellular production of ROS has been shown to lead to cellular damage and cell death. Therefore, we analyzed ROS generation in cearoin-treated cells by measuring the intensity of DCFDA dye through a ELISA reader. SH-SY5Y cells treated with different concentrations of cearoin induced the production of intracellular ROS which gradually increased with increasing doses of cearoin (Figure 4A). Because NO is also involved in the oxidative damage, we measured the NO production in SH-SY5Y cells treated with cearoin using Griess assay. The production of NO was significantly increased alongside increasing doses of cearoin (Figure 4B).

### 2.5. Cearoin-Induced Cell Death is Mediated by ROS Generation

**Figure 5:**
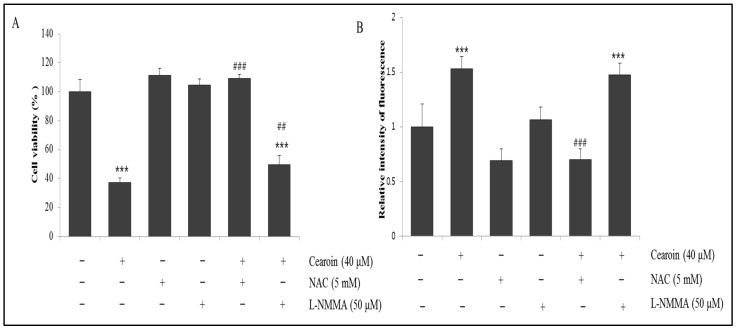

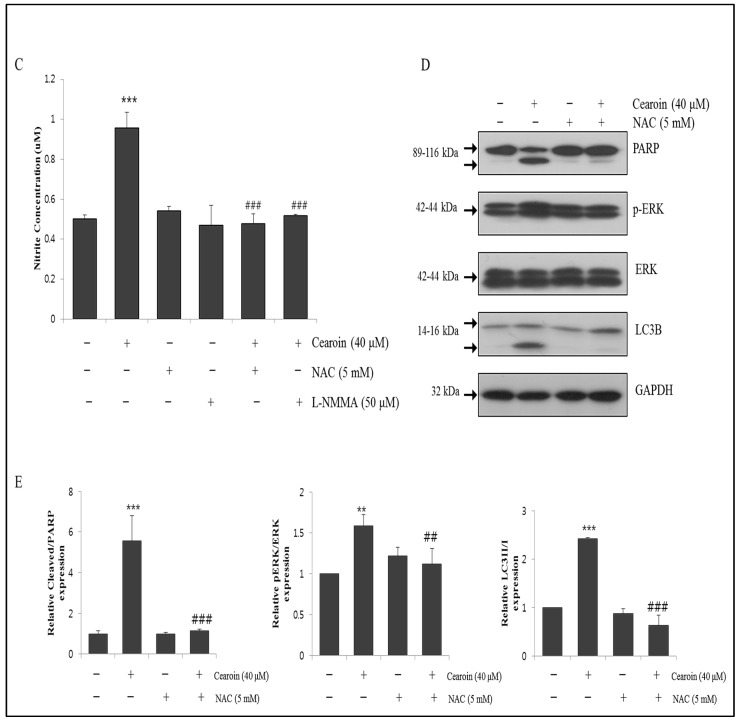
Antioxidant NAC completely reverses the cearoin-induced ERK activation, autophagy, and cell death in neuroblastoma SH SY5Y cells. Neuroblastoma SH-SY5Y cells were pre-incubated with or without ROS scavenger NAC (5 mM) or nitric oxide synthase (NOS) inhibitor L-NMMA (50 μM) for 1 h and then treated with or without cearion (40 μM) and incubated for a further 12 h. (**A**) The cell viability was measured using MTT assay; (**B**,**C**) ROS and NO levels were measured using DCFDA fluorescence method and Griess reagent respectively. Each bar represents the mean percentage alterations above or below the control (±SD) (*n =* 6). Differences were considered statistically significant at *** *p* < 0.001 as compared with the control, ## *p* < 0.01 and ### *p* < 0.001 as compared with the 40 μM cearoin treated group; (**D**) the cells were pre-incubated with or without ROS scavenger NAC (5 mM) for 1 h and then treated with or without cearion (40 μM) and incubated for a further 12 h. Then, the cells were lysed, and the protein levels of PARP, p-ERK, ERK, LC3B I/II and GAPDH were determined by Western blot analysis. The blots shown are representative of three independent experiments. GAPDH was used as a loading control; (**E**) quantification by densitometry. Each bar represents the mean fold alterations above the control (±SD) (*n* = 3). Differences were considered statistically significant at ** p < 0.01 and *** *p* < 0.001 as compared with the control, and at ## *p* < 0.01 and ### *p* < 0.001 as compared with the 40 μM cearoin treated group.

To confirm the importance of ROS and NO in cearoin-mediated cell death, a specific ROS scavenger or nitric oxide synthase (NOS) inhibitor was co-treated with cearoin in SH-SY5Y cells. Cell viability assay showed that ROS scavenger NAC significantly attenuated cearoin-induced cell death, while NOS inhibitor L-NMMA showed a significant but limited decrease in cell death induced by cearoin (Figure 5A). In the presence of ROS scavenger NAC, both ROS and NO levels induced by cearoin was inhibited (Figure 5B), while in the presence of NOS inhibitor L-NMMA, only cearoin-induced NO production was inhibited (Figure 5C). These showed that NO generation might be mediated by ROS. Furthermore, co-treatment of NAC with cearoin abolished both PARP cleavage and ERK activation, which was increased by cearoin treatment (Figure 5D). Interestingly, NAC co-treatment decreased the cearoin-induced conversion of LC3B-I to LC3B-II. From these results, we demonstrate that ROS generation mediates cearoin-induced cytotoxicity, NO production, ERK activation, and autophagy in SH-SY5Y neuroblastoma cells.

### 2.6. ERK Partially Mediates Cearoin-Induced Cell Death

**Figure 6:**
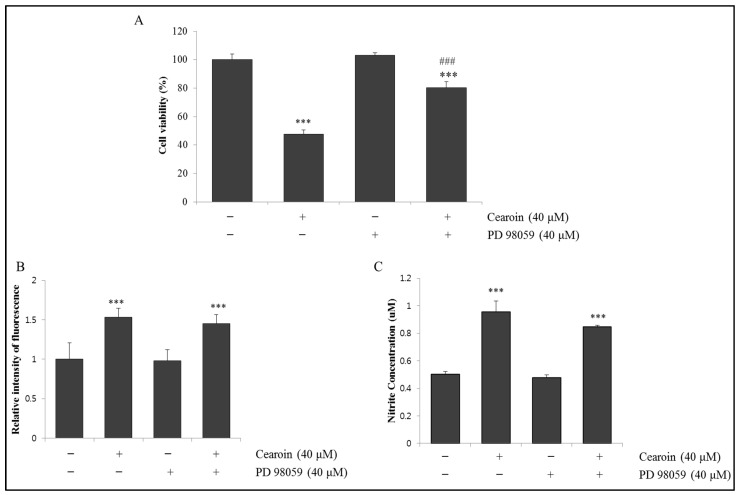
PD98059 partially reverses the cearoin-induced cell death in neuroblastoma SH-SY5Y cells. SH-SY5Y cells were pre-incubated with or without MEK inhibitor PD98059 (40 μM) for 1 h and then treated with or without cearion (40 μM) and incubated for a further 12 h. (**A**) Cell viability was measured using MTT assay; (**B**,**C**) ROS and NO levels were measured using DCFDA fluorescence method and Griess reagent respectively. Each bar represents the mean percentage alterations above or below the control (±SD) (*n* = 6). Differences were considered statistically significant at *** *p* < 0.001 as compared with the control, ### *p* < 0.001 as compared with the 40 μM cearoin treated group.

Finally, because cearoin increased the phosphorylation of ERK (Figure 2), we examined whether ERK is involved in the cearoin-induced cytotoxicity in SH-SY5Y cells. As shown in Figure 6, MEK inhibitor PD98059, which suppresses the phosphorylation of ERK, partially but significantly reversed the decreased cell viability induced by cearoin treatment (Figure 6A). ERK inhibition did not alter the generation of ROS and NO, which was induced by cearoin (Figure 6B,C). Considering that NAC abolished cearoin-induced ERK phosphorylation (Figure 5), ERK activation is one of the downstream activities derived from ROS production. Therefore, ERK inhibition did not affect the levels of ROS or NO. This data demonstrates that cearoin-induced apoptosis is partially mediated by ERK activation, which was induced by ROS generation.

## 3. Discussion

Neuroblastomas are the most common solid extracranial cancers in childhood. One-third of neuroblastoma-diagnosed children belong to a high-risk group with a drug resistance, which is attributable to extensive cellular heterogeneity [2]. Therefore, it is necessary to find novel therapeutics that can act effectively and efficiently through various anticancer mechanisms. In this study, we examined the anticancer effects of cearoin in neuroblastoma cells. We demonstrate that cearoin induces autophagy and apoptosis in SH-SY5Y neuroblastoma cells. Cearoin-induced autophagy and apoptosis is mediated by increased ROS generation. Furthermore, ERK activation via ROS production partially contributes to cearoin-induced cell death.

ROS are mostly short lived and highly reactive molecules that have a single unpaired electron in their outermost shell of electrons. ROS are radicals, including peroxides and oxygen anions, such as hydrogen peroxide, superoxide, and hydroxyl radicals. In cancer cells, ROS production is attributable to the mitochondrial dysfunction, peroxisome, metabolic activity, or receptor signaling activation [12,13]. Although low doses of superoxide or hydrogen peroxide stimulate cancer cell proliferation, disproportionate cellular ROS levels induce irreversible damages in cancer cells through cell cycle arrest and apoptosis [13]. Increased mitochondrial oxidative stress leads to cytochrome c release, caspases activation, and cell death [14]. Many chemotherapeutics have the strategy to intensely elevate intracellular ROS levels, inducing cancer cell apoptosis [15]. Cearoin treatment also significantly increased intracellular ROS and NO levels (Figure 4), which was accompanied by increased PARP cleavage and caspase-3 activity, and decreased Bcl-2 expression (Figure 3). Cearoin-induced cell death was completely reversed by ROS scavenger NAC, while it was partially recovered by NOS inhibitor L-NMMA. Although ROS scavenger NAC abrogated not only ROS but also NO, NOS inhibitor L-NMMA abolished only NO, not ROS. Compared to L-NMMA, NAC significantly reversed the cytotoxicity increased by cearoin. Therefore, the NAC-induced decrease in cytotoxicity might be mainly attributable to scavenging ROS. These data suggest the critical role of ROS in cearoin-induced anticancer effects. In addition, NO production might be due to the ROS generation, because NAC abolished NO production. It has been reported that ROS mediates NO production through ERK/JNK signaling pathway [16]. Because NAC has been known to modulate the expression of genes for many signaling molecules, it should be considered the indirect effects of NAC. Therefore, further studies will be needed to clarify this issue.

Generally, the ERK pathway activated by growth factors and K-ras has been known to induce cell proliferation in cancer [17]. However, there are several reports showing that ROS-dependent ERK activation triggers cell cycle arrest and apoptosis in cancer cells. Anticancer agents such as cisplatin, or etoposide require prolonged ERK activation for inducing apoptosis in various immortalized or transformed cells [18]. Consistent with in vitro results, ROS-mediated activation of the ERK pathway inhibited pancreatic tumor growth in a xenograft model [19]. In our study, cearoin increased ERK phosphorylation and the inhibition of ERK using PD98059 partially reversed the cell death effect of cearoin (Figure 6). These data suggest that ROS-induced ERK activation is one of the molecular mechanisms required for cearoin-mediated anticancer effects in SH-SY5Y neuroblastoma cells. Considering that the ERK inhibitor could not able to inhibit the ROS and NO production induced by cearoin, ERK activation might be the downstream signaling of ROS production.

Autophagy is a highly conserved catabolic process that recycles damaged cellular components in response to various stresses such as nutrient deprivation or viral infection. It is morphologically characterized by the formation of double membrane autophagosomes. Autophagy is a double-sided cellular program, because it triggers either cell death or survival. In case of extensive damages, autophagy promotes another programmed cell death pathway, known as autophagic cell death [20]. Interestingly, cearoin-induced autophagy was inhibited by NAC co-treatment (Figure 5D). It has been reported that several anticancer agents such as resveratrol [21] or cucurbitacin [22] induce autophagy through ROS production. Previous reports have suggested the several molecular mechanisms by which ROS induce autophagy. One of the mechanisms is that ROS oxidize ATG4 leading to the increased autophagosome formation [23]. On the other hand, apogossypolone disrupted the interaction of Beclin-1 and Bcl-2, which is accompanied by the increased ROS-mediated autophagy [24]. Therefore, cearoin might also increase the oxidation of ATG4 or interrupt the interaction of Beclin-1 and Bcl-2, thereby inducing autophagy activation. Further study will be needed to clarify this issue.

In this study, we demonstrate for the first time that cearoin induces apoptosis. There are several researches showing the anticancer effects of some compounds with benzophenone structure. Injection of benzophenone semicarbazone inhibited cell growth, decreased tumor weight, and increased mean survival time in Swiss albino mice bearing ehrlich ascites carcinoma cells [25]. A benzophenone 7-epiclusianone isolated from *Garcinia brasiliensis* decreased the S and G2/M populations, increased the number of cells at sub-G1, and increased apoptotic cell death in glioblastoma cell lines [26]. In this regard, cearoin might be newly included in the anticancer compounds with benzophenone structure.

In conclusion, we demonstrate for the first time that cearoin has anticancer effects in neuroblastoma SH-SY5Y cells. Cearoin leads to autophagy and apoptosis through the increased ROS generation. Furthermore, cearoin-induced cell death is partially mediated by ROS-dependent ERK activation.

## 4. Materials and Methods

### 4.1. Materials and Materials

Cearoin was isolated from the ethanol extract of dried heartwoods of *D. odorifera* as described previously [27]. Cearoin stock solution was prepared in DMSO and stored at −20 °C, which was diluted using serum-free media during the treatment. Similarly, stock solution of anticancer drug cyclosphosphamide was prepared in DMSO and diluted in serum-free media during treatment. Dulbecco’s Modified Eagle’s Medium (DMEM) and all medium additives were obtained from Gibco BRL. Cyclophosphamide, Sodium nitrite, *N*-Acetyl-l-cysteine (NAC), NG-methyl-l-arginine acetate (L-NMMA), 3-(4,5-dimethylthiazol-2-yl)-2,5-diphenyl tetrazolium bromide (MTT), and 2-7-dichlorodihydrofluorescein diacetate (DCFDA) fluorescence kit were purchased from Sigma Aldrich (St Louis, MI, USA). PD98059 was purchased from Calbiochem (La Jolla, CA, USA). Caspase-3 activity kit was purchased from Biovison (Milpitas, CA, USA). The antibodies specific for α-spectrin, PARP, LC3B, p-ERK, ERK, p-JNK and JNK were purchased from cell signaling Technology (Danvers, MA, USA). The β-actin was purchased from Santa Cruz Biotechnology (Santa Cruz, CA, USA) and GAPDH was purchased from Thermo Fisher (Illinois, Rockford, AL, USA).

### 4.2. Cell Culture

The human neuroblastoma cell line, SH-SY5Y, was purchased from the Korean Cell Line Bank and cultured in Dulbecco’s Modified Eagle’s Medium (Gibco BRL, Grand Island, NE, USA) supplemented with 10% fetal bovine serum in a 5% CO_2_ atmosphere at 37 °C. The cells were seeded and treated with various doses of cearoin in serum-free media 24 h later.

### 4.3. MTT Assay

Cell viability was calculated by the reduction of MTT. Human neuroblastoma SH-SY5Y cells were seeded at 2 × 10^5^ cells per well in a 96-well plate and treated with various concentrations of cearoin for 6 h or 12 h. In the case of NAC, L-NMMA, or PD98059 co-treatment, the cells were pre-treated with 5 mM NAC or 50 μM L-NMMA or 40 μM PD98059 for 1 h, followed by cearoin treatment for 12 h. After 12 h incubation the media was removed gently and 100 μL MTT solution (1 mg/mL) prepared in serum-free media was added to each well. The plates were then incubated at room temperature for 2 h and reduced purple blue MTT formazan crystals were solubilized by adding 100 μL DMSO to each well and shaking in a rocker for 15 min. The absorbance was then measured at 540 nm using a micro-plate ELISA reader.

### 4.4. Measurement of Intracellular ROS

Intracellular ROS was measured by using DCFDA fluorescence dye. SH-SY5Y cells were seeded at 2 × 10^5^ cells per well in a black, clear bottom 96-well plate and incubated for 24 h. Then cells were treated with cearoin in concentrations of 5, 10, 20, 40, 80 or 160 μM for 12 h. In the case of NAC, L-NMMA, or PD98059 co-treatment, the cells were pre-treated with 5 mM NAC or 50 μM L-NMMA or 40 μM PD98059 for 1 h, followed by cearoin treatment for 12 h. For positive control, H_2_O_2_ was treated at the last 45 min of treatment. Then DCFDA dye was treated in 20 μM concentration for 20 min using serum-free media. At the end of incubation, the cells were washed with PBS and an intensity of fluorescence was measured at wavelength 485 nm for excitation and 535 nm for emission in a fluorescence micro-plate ELISA reader.

### 4.5. Measurement of NO Production

NO generation was determined by measuring NO_2_^−^ concentrations in the culture supernatant using Griess reagents. SH-SY5Y cells were seeded at 0.5 × 10^6^ cells per well in a 12-well plate and incubated for 24 h. Then cells were treated with cearoin in concentrations of 5, 10, 20, 40, 80 or 160 μM for 12 h. In the case of NAC, L-NMMA, or PD98059 co-treatment, the cells were pre-treated with 5 mM NAC or 50 μM L-NMMA or 40 μM PD98059 for 1 h, followed by cearoin treatment for 12 h. Culture supernatant (50 μL) from each group was mixed with 50 μL of the Griess reagent (mixture in 1:1 ratio of 0.1% *N*-1-napthylethylenediamine dihydrochloride in water and 1% sulphanilamide in 5% phosphoric acid) and then incubated for 10 min. The optical density was measured at 540 nm. NO_2_^−^ concentrations were determined from a standard curve prepared with known concentrations of NaNO_2_.

### 4.6. Caspase-3 Activity Assay

Activation of caspase-3 was measured by using caspase-3/CPP32 colorimetric assay kit (BioVision, Milpitas, CA, USA). The SH-SY5Y cells were cultured in a 6-well plate and incubated for 24 h. The cells were treated with cearoin in different doses and incubated for 3 h. Then, caspase-3 activity was measured according to the manufacturer’s instructions.

### 4.7. Western Blot Analysis

Human neuroblastoma SH-SY5Y cells were seeded at 2 × 10^6^ cells per well in a 6-well plate and treated with various concentrations of cearoin for 12 h. In the case of NAC, L-NMMA, or PD98059 co-treatment, the cells were pre-treated with 5 mM NAC or 50 μM L-NMMA or 40 μM PD98059 for 1 h, followed by cearoin treatment for 12 h. The cells were then washed twice with ice cold PBS and lysed in RIPA buffer (Biosesang, Seoul, Korea) containing 50 mM Tris-HCl PH 7.5, 150 mM NaCl, 1% triton X-100, 1% Sodium deoxycholate, 0.1% SDS, 2 mM EDTA PH 8.0 and supplemented with 1% protease inhibitor cocktail and phosphatase inhibitors (Roche Life Science, Mannheim, Germany). The cell lysates were centrifuged at 15,000 rpm for 10 min at 4 °C and the supernatants were collected and used for Western blot analysis. Protein concentrations were measured using BCA assay kit (Thermo Scientific, Illinois, Rockford, AL, USA). Equal amount of proteins from each sample were subjected to SDS-PAGE and transferred to PVDF membrane (EMD Millipore, Bedford, MA, USA). The membranes were incubated in rocking mode for 1 h in blocking solution i.e., 5% skim milk prepared in T-TBS (TBS buffer containing 1% tween 20) at RT (Room Temperature) and incubated overnight at 4 °C with specific antibodies, which were diluted (1:1000) in 5% skim milk. After that, the membranes were incubated with anti-rabbit and anti-mouse IgG horseradish peroxidase-linked secondary antibodies for 1 h at room temperature. Then membranes were washed thrice for 5 min with T-TBS, which were later developed in film using a chemiluminescent mixture (Thermo Scientific). Then the expression levels were quantified by densitometry using ImageJ software (National Institutes of Health, Bethesda, MA, USA).

### 4.8. Statistical Analysis

All experiments were performed at least three times using independent experiments with identical results. All results are expressed as means ± standard deviation (SD) and the presented figures are representative of the series of experiments. Statistical analyses were performed using GraphPad Prism software where the significance of difference was determined using one-way ANOVA and turkey tests for all paired data. A value of *p* < 0.05 was considered significant.
